# Effect of Graphene Addition on the Thermal and Persistent Luminescence Properties of Gd_2.994_Ce_0.006_Ga_3_Al_2_O_12_ and Gd_2.964_Ce_0.006_Dy_0.03_Ga_3_Al_2_O_12_ Ceramics

**DOI:** 10.3390/ma15072606

**Published:** 2022-04-01

**Authors:** Daniela Kujawa, Daria Szewczyk, Vitalii Boiko, Damian Bęben, Paweł Głuchowski

**Affiliations:** 1 Institute of Low Temperature and Structural Research PAS, PL 50422 Wroclaw, Poland; d.kujawa@intibs.pl (D.K.); d.szewczyk@intibs.pl (D.S.); v.boiko@intibs.pl (V.B.); d.beben@intibs.pl (D.B.); 2 Nanores, PL 51317 Wroclaw, Poland

**Keywords:** garnet, cerium, dysprosium, ceramics, graphene, persistent luminescence, thermal conductivity

## Abstract

The gadolinium, gallium, aluminum garnet doped with cerium and co-doped with dysprosium ions were prepared using sol gel method. The SEM images show that after synthesis, the grains are below 100 nm. The powders were ultrasonically mixed with graphene nanoflakes and ceramics were prepared using the high pressure low temperature sintering technique. A series of the ceramics was prepared using different graphene content. The structure of the samples was examined using X-ray diffraction (XRD), scanning electron microscope (SEM) and Raman techniques. The spectroscopic properties were checked using conventional and persistent luminescence spectra measurements. The thermoluminescence glow curves and fading time of persistent luminescence measurements were performed to check how the graphene presence affects the electron traps number and depth. It was found that the addition of graphene improved the thermal conductivity of co-doped samples. This resulted in faster release of deeper traps and an increase in fading of persistent luminescence. The possibility of releasing energy from deep traps without additional stimulation may allow the use in different applications, the matrices and luminescent ions, which so far did not show persistent luminescence at room temperature.

## 1. Introduction

Persistent luminescence is a phenomenon where material emits light for a long time after ceasing the irradiation. This type of luminescence as an irradiation source can be used for visible light, gamma, X-ray or UV radiation. The long-time luminescence decay is the effect of releasing the electrons from the traps with the help of thermal energy [1]. The energy needed for releasing the traps is an important factor that has a great impact on the intensity and time of persistent luminescence. Therefore, many attempts are made to prepare materials with the appropriate energy gap and electronic structure, where defects/traps are located near the conduction band (CB) and the energy of room temperature is sufficient to transfer the electron from the traps to CB. For this reason, new matrices and various dopants are used to develop more efficient persistent luminescence phosphors [2]. Another approach to reducing the energy needed to release charges from traps may be to increase the thermal conductivity of the matrix, so that less energy would be needed to release energy from deeper traps.

Different additives can increase thermal conductivity of the composites, but the most well-known and recently used is graphene. The allotropic form of carbon, called graphene, consists of one plain layer of carbon atoms that are organised in a honeycomb lattice. In this structure, the low-lying electrons behave like massless relativistic Dirac fermion, which are the cause of many interesting properties such as conductance quantization feasibilities of inducing a band gap through the lateral quantum confinement and unusually high room temperature carrier mobility. One of the most interesting properties of graphene is its large thermal conductivity. The diamond-like carbon value of thermal conductivity is about 1.7 W/mK, and for graphene this value increases to 5300 W/mK [3]. For this reason, graphene is used as an additive to enhance thermal conductivity of different composites where the matrix is a polymer [4,5] or metal [6,7].

Garnets are the most common materials used for lightening or various optical applications due to their important physical properties such as good thermal conductivity, large thermal stability and optical isotropy. In recent years, two of the most widely used garnets are yttrium aluminium garnet (Y_3_Al_5_O_12_) [8] and Lu_3_Al_5_O_12_ (LuAG) [9,10,11,12,13,14,15], because of their unique radiation conversion ability and wide energy band gap. Different types of garnet can be optically active by replacing Y^3+^/Gd^3+^ ions by other lanthanide ions (e.g., Ce^3+^, Nd^3+^, Er^3+^, Tb^3+^, Eu^3+^) [16,17,18]. It is possible because both kinds of ions have similar chemical properties and ionic radii [19]. For example, cerium doped Y_3_Al_5_O_12_ emits in the green/yellow range after excitation with blue radiation. The most desirable result is to obtain perfectly white light, so we need to use a phosphor, which has a wide emission band covering green and red regions after a blue light excitation. By doping the garnet lattice with some cations, we can modulate the emission colour. The smaller ions shift the emission band towards blue, and larger ones shift the emission band towards red [20]. Gd_3_Ga_3_Al_2_O_12_ garnet (GGAG), doped with Ce^3+^, focuses the attention of researchers due to the possibility of using this matrix as a good quality scintillator unique light yield where energy resolution can reach up to 50,000 photons/MeV. Because of the strong crystal field splitting, the emission of Ce^3+^ doped GGAG has a maximum of about 565 nm and can be used for warm white LEDs [21,22].

This work was conducted to find out whether the addition of graphene flakes will change the thermal conductivity of the ceramics and in consequence their persistent luminescence properties. It was checked whether the modification of the thermal conductivity has an impact on the release of electrons from shallow and/or deep energy traps. Better thermal conductivity should reduce the amount of energy needed to release charges from energy traps and thus modify the properties of persistent luminescence. The ability to control persistent luminescence is of great importance in many applications. First of all, such emission is currently used in emergency marking and bioimaging. For such materials, it is important to obtain emissions in the red or infrared range. Unfortunately, some matrices and luminescent ions that could be useful here have traps too deep to observe emission at room temperature. In order to allow their emptying and to observe luminescence at the same time, various actions were taken that could release the trapped energy without additional stimulation. To verify our assumption, ceramics showing persistent luminescence and composites containing an admixture of graphene flakes were prepared and characterized. For all materials, their structure, morphology, spectroscopic properties, thermoluminescence and thermal conductivity were examined. Based on the obtained results, the mechanism of persistent luminescence was constructed, taking into account the impact of the graphene flakes addition and its relationship with the thermal conductivity of composites.

## 2. Materials and Methods

### 2.1. Materials

The gadolinium oxide (Gd_2_O_3_, powder, 99.995%, Stanford Materials Corporation, Lake Forest, IL, USA), dysprosium oxide (Dy_2_O_3_, powder, 99.99%, Stanford Materials Corporation), hydrated gallium nitrate (Ga(NO_3_)_3_∙xH_2_O, Onyxmet, Olsztyn, Poland), aluminium nitrate nonahydrate (Al(NO_3_)_3_∙9H_2_O, 98%, Alfa Aesar, Haverhill, MA, USA) and cerium nitrate hexahydrate (Ce(NO_3_)_3_∙6H_2_O, 99,99%, Sigma Aldrich, Saint Louise, MO, USA), citric acid (99.5%, Alfa Aesar) and ethylene glycol (96%, POCH Basic, Gliwice, Poland) were used to synthesize garnet powders.

### 2.2. Preparation

The powders were prepared by the Pechini sol-gel method [23]. The first stage of the synthesis was the three-fold recrystallization of oxides after dissolution in the nitric acid (65%, POCH Basic). After the last evaporation of water, the heating was switched off. Then, distilled water and the stoichiometric amount of gallium, aluminium and cerium nitrates were added and left on a magnetic stirrer until all the nitrates were dissolved. An appropriate amount of citric acid (CA) and ethylene glycol (EG) were sequentially added (5 M of CA and EG on 1 M of cations) and stirred until a clear solution was obtained. After dissolving all the reagents, the solution was placed in a dryer at 90 °C for approximately 7 days. In that way the obtained brown resin was calcined at 1100 °C for 8 h. The resulting powder was grounded in the agate mortar. The mixtures of garnets and graphene flakes were made using different concentration (0.1, 0.2, 0.4, 0.8 and 1.6 weight-%) of commercial single layer graphene (ACS Materials). The quality of commercial graphene was confirmed using XRD, Raman and TEM techniques (Supplementary Figures S1–S3). To the mixture of the garnet powder and graphene was added acetone and the solution was dispersed using high power ultrasonic homogenizer (Tefic Biotech Co., TF-900N, 900 W, Process Time—5 min, Pulse ON 3 s, Pulse OFF 3 s, Power 70%) and then evaporated. The resulting mixture was finely ground in the agate mortar for 30 min and taken for ceramic preparation. The ceramics were prepared using a low temperature high pressure method [24]. Shortly, the pellets were formed from the powder at room temperature and 200 MPa pressure. Then the pellets (green body) were placed inside a graphite heater separated from the pellets by a boron nitride layer. The heater was fixed in the toroid shaped container. A quasi-isostatic pressure of 8 GPa was applied using the axial pressure of two anvils. After reaching the desired pressure, the pellet was sintered at 500 °C for 1 min. After the sintering process, the ceramics were polished using grinding paper. It should be noted that the graphene used in the production of ceramics, despite the application of high pressure, still retains most of the properties of the starting material, not graphite, which was shown by us earlier [25].

### 2.3. Characterization

The XRD were collected between 10 and 60 2θ degrees at room temperature by an X’PERT PRO PANalytical (Malvern Pananalytical, Malvern, UK) diffractometer using CuKα_1_ radiation (1.5406 Å) and step 0.03°. The morphology and homogeneity of the samples were checked using SEM images taken using FEI Helios G4 PFIB CXe DualBeam microscope (Thermo Fischer Scientific, Freiberg, Germany). All measured samples were placed on aluminum holders using copper tape. The elemental composition was determined and mapped using an EDS detector: Bruker Nano GmbH XFlash Detector 630 (Bruker, Billerica, MA, USA). The excitation spectra of Gd_3_Ga_3_Al_2_O_12_ doped with cerium and co-doped with dysprosium were measured using FLS980 Fluorescence Spectrometer from Edinburgh Instruments (Livingston, UK) equipped with a holographic grating of 1800 lines/mm, flashed at 300 mm focal length monochromators in Czerny Turner configuration. To obtain the excitation and emission spectra a 450 W Xenon lamp was used and as a detector the R928P side window photomultiplier tube from Hamamatsu (Hamamatsu Photonics, Shizuoka, Japan) was used. The persistent luminescence (PersL) spectra of the GGAG were measured at room temperature after 5 min laser diode irradiation (CNI Lasers, Changchun, China) with a 445 nm wavelength using SilverNova CCD spectrometer (StellarNet Inc., Tampa, FL, USA) with 200 µm slit and 1 s integration time. The same system was used for conventional luminescence measurements. The thermoluminescence (TL) and PersL decay curves were measured using a lexsygresearch TL/OSL reader (Freiberg Instruments GmbH, Freiberg, Germany). The signal was detected by a R13456PMT detector (Hamamatsu Photonics, Shizuoka, Japan) monitoring emission in whole spectral range (from 185 to 980 nm) with an integration time of 0.1 s. The TL curve was registered from room temperature to 300 °C. As an irradiation source, a blue laser diode PL 450B (λ_max_ = 450 nm, FWHM  =  2 nm, P = 1 mW/cm^2^) was used. Before measurements, each sample was heated to 350 °C and kept for 1 min to traps cleaning. Thermal conductivity measurements were carried out using Physical Property Measurement System (PPMS^®^) operating in the Thermal Transport Option. The temperature range was from 2 K up to 295 K, the thermal conductivity coefficient was determined in a continuous mode of the device with an accuracy of ±5%.

## 3. Results and Discussion

### 3.1. Structure and Morphology Characterization

**Figure 1:**
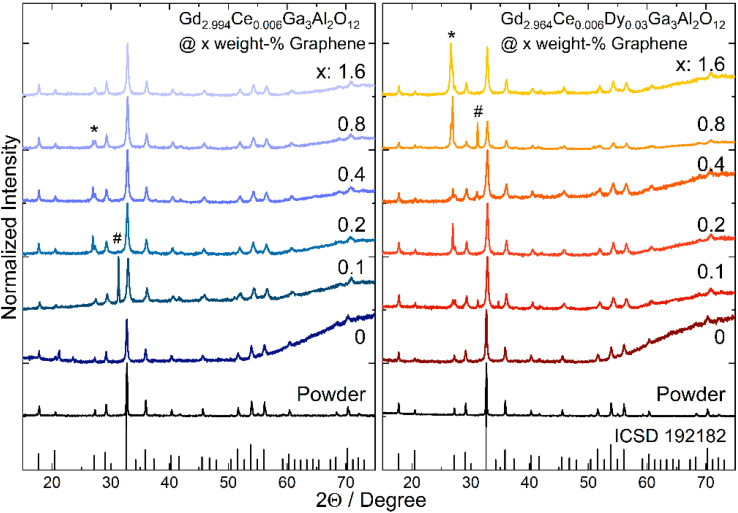
XRD patterns of Gd_2.994_Ce_0.006_Ga_3_Al_2_O_12_ and Gd_2.964_Ce_0.006_Dy_0.03_Ga_3_Al_2_O_12_ powders and ceramics with different graphene content (*—graphite, #—abrasive MgAl_2_O_4_).

The XRD diffraction technique was used to study the structure of the Gd_2.994_Ce_0.006_Ga_3_Al_2_O_12_ and Gd_2.964_Ce_0.006_Dy_0.03_Ga_3_Al_2_O_12_ powders and ceramics (Figure 1). Resulted XRD patterns match well with the cubic structure of Gd_3_Ga_3_Al_2_O_12_. Two peaks are observed in the XRD patterns of the ceramics that are not assigned to the garnet structure. For the ceramics doped only with cerium with the addition of 0.2, 0.4 and 0.8 weight-% of graphene and for all ceramics co-doped with dysprosium with graphene addition, one peak appeared at 26.8 degrees (marked with * in the XRD patterns). This peak is assigned to the graphite structure whose presence is related to the effect of high pressure on the graphene flakes [25]. The absence of this peak in part of the ceramics is caused probably by aggregation of the graphene pellets inside the ceramics and its lower content on the surface. This is also confirmed by EDS studies that show non homogenous distribution of the graphene on the surface and between grain boundaries. Another peak that has been observed for 2Θ = 31.2 (marked with #) is assigned to spinel (MgAl_2_O_4_) [26], which is a natural mineral used as an abrasive material. As the ceramics after sintering have a rough surface, they were polished and part of the spinel may remain on the surface of the ceramics. Broadening of the peaks may be observed for all ceramic samples that are related to the strains introduced into the structure and decrease in the grains caused by decomposition of the nanograins surface during high pressure sintering. It is also interesting to note, that for the ceramics with graphene addition, the diffraction peaks are shifted towards higher angles. This could be an effect of the reduction atmosphere during sintering in the presence of graphene (carbon). It may bind oxygen atoms from the compound and slightly disturb the stoichiometry of the garnet structure. In consequence it may also have an impact on the spectroscopic properties of the Gd_2.994_Ce_0.006_Ga_3_Al_2_O_12_@xGraphene and Gd_2.964_Ce_0.006_Dy_0.03_Ga_3_Al_2_O_12_@xGraphene ceramics.

**Figure 2:**
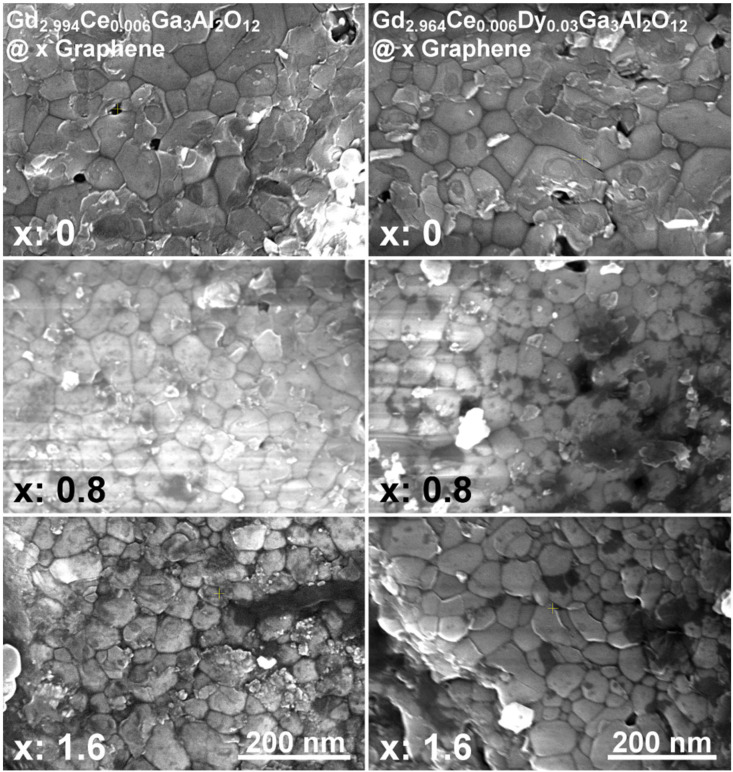
SEM images of Gd_2.994_Ce_0.006_Ga_3_Al_2_O_12_ and Gd_2.964_Ce_0.006_Dy_0.03_Ga_3_Al_2_O_12_ ceramics with different graphene content.

The representative SEM images of the Gd_2.994_Ce_0.006_Ga_3_Al_2_O_12_ and Gd_2.964_Ce_0.006_Dy_0.03_Ga_3_Al_2_O_12_ ceramics with different graphene content are shown in Figure 2. It can be seen that the grains are well sintered and keep the nanosized character, which also has an impact on their properties. In a few places, single pores can be seen, which are the remnants of the aggregates of powder used for ceramic sintering. There are also clear black, carbon spots visible in the images taken for ceramics containing graphene. Unfortunately, it can also be seen that graphene is not uniformly distributed in all places.

**Figure 3:**
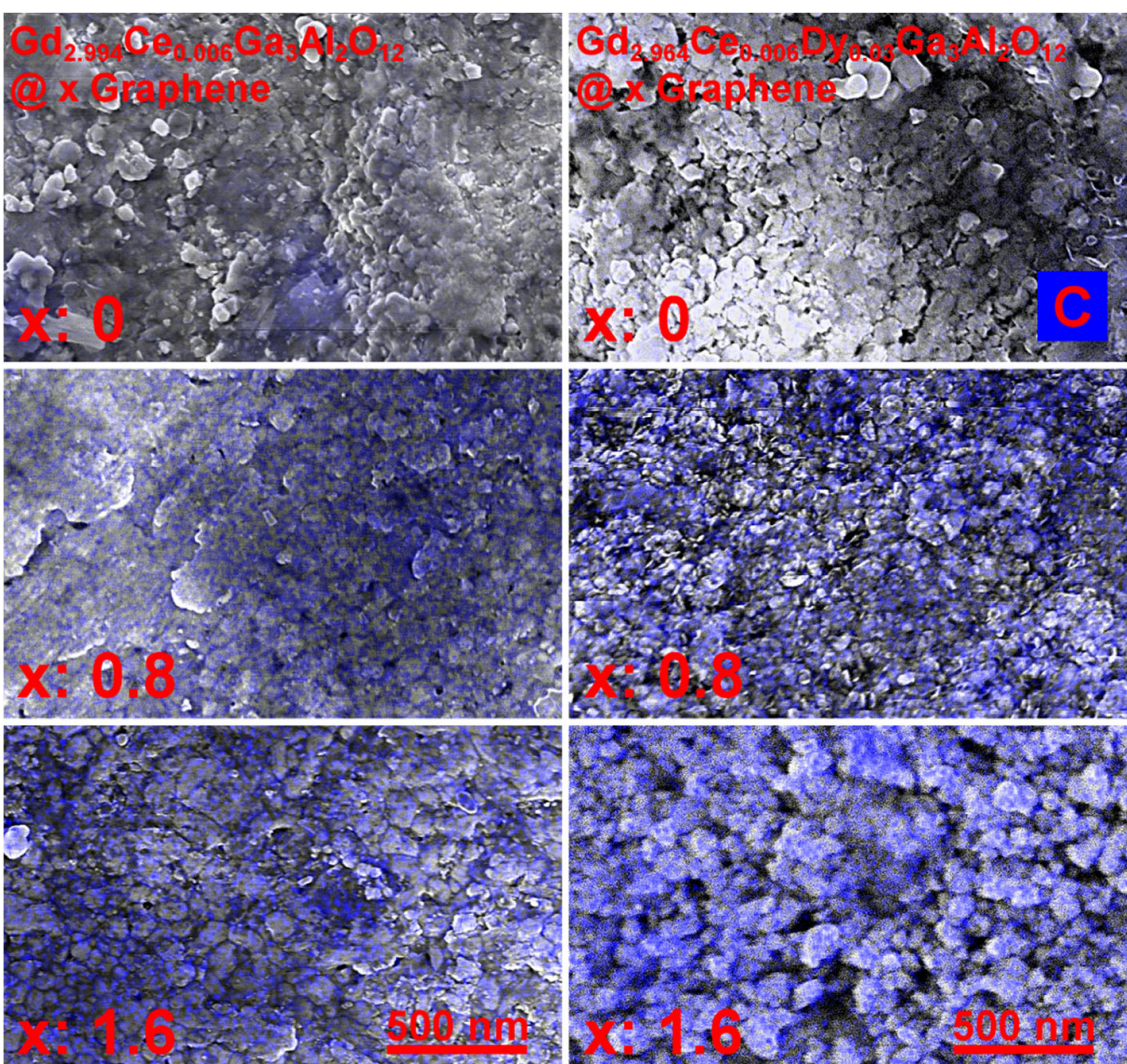
SEM + EDS maps indicating carbon (C) amount in the Gd_2.994_Ce_0.006_Ga_3_Al_2_O_12_ and Gd_2.964_Ce_0.006_Dy_0.03_Ga_3_Al_2_O_12_ ceramics with different graphene content.

To check how the graphene (carbon—“C”) is distributed through the ceramics volume, the EDS maps of the Gd_2.994_Ce_0.006_Ga_3_Al_2_O_12_ and Gd_2.964_Ce_0.006_Dy_0.03_Ga_3_Al_2_O_12_ ceramics were taken (see Figure 3). It can be seen that the graphene flakes are located on the grains and in the grain boundaries, but they do not always form continuous connections, which may result in a greater dissipation of heat at the grain boundaries, and its worse distribution throughout the entire volume of the ceramic. This may also affect the transport of electrons between individual grains, and, consequently, less efficient energy transfer in individual crystallites.

### 3.2. The Excitation and Photoluminescence Spectra of Gd_2__.994_Ce_0.006_Ga_3_Al_2_O_12_ and Gd_2.964_Ce_0.006_Dy_0.03_Ga_3_Al_2_O_12_ Ceramics with Different Graphene Content

**Figure 4:**
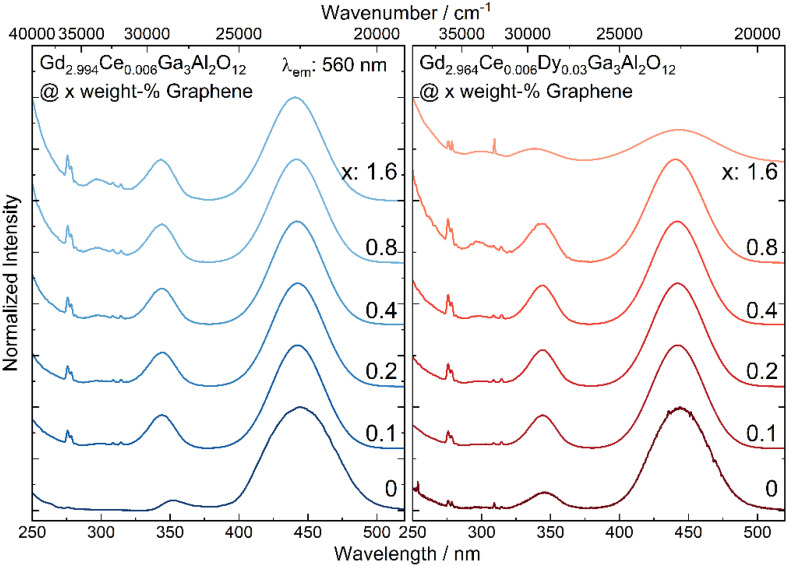
The excitation spectra of Gd_2.994_Ce_0.006_Ga_3_Al_2_O_12_ and Gd_2.964_Ce_0.006_Dy_0.03_Ga_3_Al_2_O_12_ ceramics with different graphene content.

The excitation spectra of Gd_2.994_Ce_0.006_Ga_3_Al_2_O_12_ and Gd_2.964_Ce_0.006_Dy_0.03_Ga_3_Al_2_O_12_ nanoceramic samples with different graphene content show two broad bands at 344 and 441 nm (29,100 and 22,650 cm^−1^) observed due to 4f-5d_2_ and -5d_1_ transitions of Ce^3+^ and sharp peaks at 275 and 310 nm (36,360 and 32,260 cm^−1^) assigned to transitions from ^6^I_J_ and ^6^P_J_ excited level to the ^6^S_7/2_ ground state of Gd^3+^, respectively (Figure 4). For all spectra, the 5d bands were fitted to check if the graphene admixture has an impact on the crystallographic environment of the luminescent ions. It is known that an increase in the 5d states splitting (Δ_21_) and their redshift is a result of a higher disorder of the surrounding of Ce^3+^ ions and displacement from the cubic polyhedron to disordered square anti-prism (dodecahedron) [27]. Furthermore, Dorenbos has shown [28] that the shift of the 5d excitation bands in the garnet family is proportional to the crystal field splitting caused by tetragonal distortion. For the ceramics containing graphene a blue shift of the 5d_2_ and redshift for 5d_1_ levels was observed (Table 1). The distance between maxima of the 5d_2_ and 5d_1_ bands (Δ_21_) increases when the graphene concentration increases. It was also observed that full width at half maximum (FWHM) of the bands for the pure ceramic are broader in case of 5d_1_ and shallower in case of 5d_2_ level compared to the samples with graphene addition. A large value and a deviation from the FWHM change tendency observed for the sample co-doped with Ce^3+^/Dy^3+^ with the highest concentration of graphene (1.6%), is related to the fact that it was difficult to register the excitation spectrum for this sample with the same slits as for other samples and therefore in this case they were opened wider. It can also be noticed that FWHM of 5d_1_ decreases with an increase in graphene content and for the 5d_2_ level the tendency is the opposite. As in the GGAG matrix, the optically active ions may occupy several positions [29], the fact of the bands width decreasing with an increase in the graphene content suggests that emission from a particular crystallographic site begins to dominate. Consequently, this can lead to a lower excitation efficiency of the ions in different sites.

**Figure 5:**
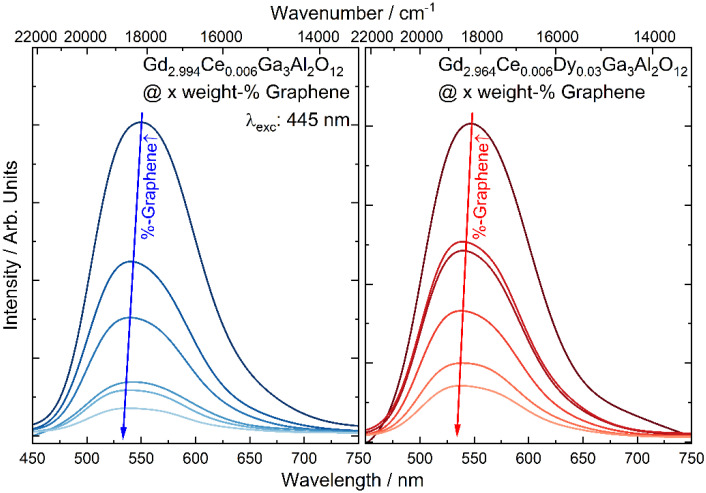
The emission spectra of Gd_2.994_Ce_0.006_Ga_3_Al_2_O_12_ and Gd_2.964_Ce_0.006_Dy_0.03_Ga_3_Al_2_O_12_ ceramics with different graphene content.

The emission spectra of the Gd_2.994_Ce_0.006_Ga_3_Al_2_O_12_ nanoceramics doped and co-doped with cerium and dysprosium ions were measured under 445 nm laser diode excitation (Figure 5). An intense broad band centered at 550 nm, associated with the transitions from the 5d_1_ level to the ^2^F_5/2_ ground state of Ce^3+^ [30] can be observed for all samples. One can notice that with an increase in the graphene content, the emission band is shifted toward a lower wavelength. The shift of the luminescence band is an effect of changes in the Ce^3+^ environment resulting from alternation of the type of coordination and bond length between the luminescent ion and surrounding ligands [31,32]. Herrmann et al. [33] showed that Ce^3+^ emission peak positions shift towards longer wavelengths while the Ce^3+^/Ce^4+^ ratio decreases. In the case of Gd_2.994_Ce_0.006_Ga_3_Al_2_O_12_ and Gd_2.964_Ce_0.006_Dy_0.03_Ga_3_Al_2_O_12_ nanoceramics the addition of a redox agent (carbon in form of graphene) may lead to a reduction in residual Ce^4+^ to Ce^3+^ and, as a result, a shift of the emission wavelength toward lower wavelengths. The decrease in the emission intensity is related to the dark gray color of the ceramics after the addition of graphene and their strong light absorption.

### 3.3. Persistent Luminescence of Gd_2.994_Ce_0.006_Ga_3_Al_2_O_12_ and Gd_2.964_Ce_0.006_Dy_0.03_Ga_3_Al_2_O_12_ Ceramics with Different Graphene Content

**Figure 6:**
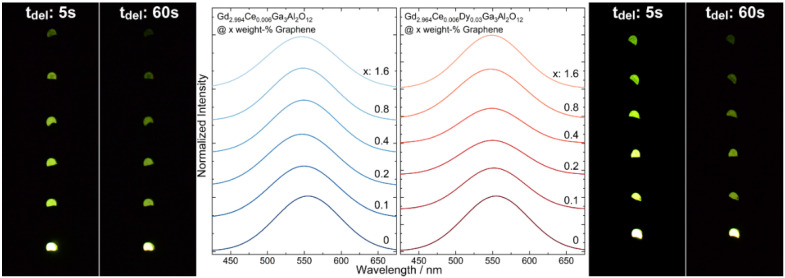
Persistent luminescence spectra taken seconds after ceasing 445 nm irradiation and photo of Gd_2.994_Ce_0.006_Ga_3_Al_2_O_12_ and Gd_2.964_Ce_0.006_Dy_0.03_Ga_3_Al_2_O_12_ ceramics with different graphene content.

Persistent luminescence spectra of Gd_2.994_Ce_0.006_Ga_3_Al_2_O_12_ and Gd_2.964_Ce_0.006_Dy_0.03_Ga_3_Al_2_O_12_ ceramics were registered after ceasing 445 nm laser diode irradiation (Figure 6). The persistent luminescence spectra were analyzed and the results are presented in Table 2. On their basis, it was found that maximum intensity of persistent luminescence depends of the concentration of graphene. Similar to the conventional luminescence, after the addition of graphene, a blue shift of the spectrum maximum was observed with only two exceptions: Gd_2.994_Ce_0.006_Ga_3_Al_2_O_12_ with 0.2 weight-% and Gd_2.964_Ce_0.006_Dy_0.03_Ga_3_Al_2_O_12_ with 1.6 weight-% of graphene. The FWHM is changed depending on the sample composition. For garnets doped only with cerium the FWHM decreases compared to the ceramic without graphene addition but the changes are irregular. In the case of the ceramics co-doped with dysprosium, FWHM increased in comparison to the pure one and simultaneously decreased with the increase in the amount of graphene.

**Figure 7:**
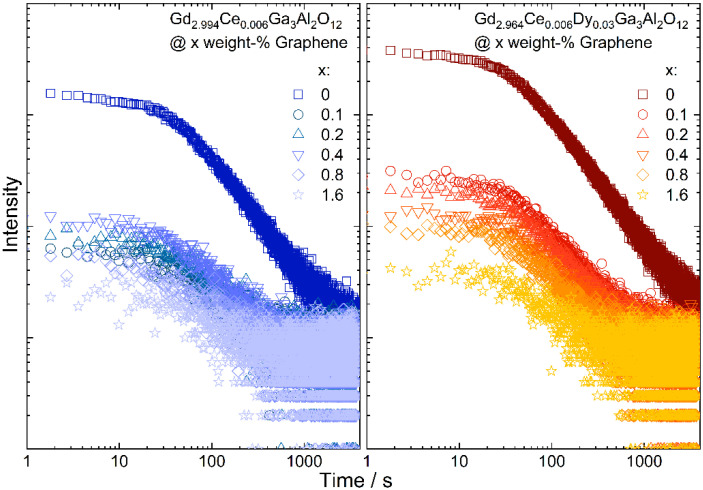
Persistent luminescence fading observed in Gd_2.994_Ce_0.006_Ga_3_Al_2_O_12_ and Gd_2.964_Ce_0.006_Dy_0.03_Ga_3_Al_2_O_12_ ceramics with different graphene content.

To check how the graphene addition affects the persistence luminescence fading, the samples were continuously excited with a 450 nm laser diode for 5 min and after ceasing irradiation, the intensity of the persistent luminescence in the function of time was measured (Figure 7). The initial luminescence intensity was lower for samples with graphene, which was expected, as the ceramics were grey and black graphene flakes absorb the yellow emission from the Ce^3+^. The persistent luminescence intensity was higher for the ceramics co-doped with Dy^3+^ ions. For all samples, emission intensity decreases with the increase in graphene content. For all samples after 30 min of measurement, the intensity was similar. The decay of the emission intensity was fitted using a biexponential formula. The short component (τ_1_) of the decay was assigned to the bright emission in the initial time, and was observed due to releasing of the electrons from the shallow traps. As the carriers from deeper traps need more energy to be released, they are transferred more slowly to the excited states of Ce^3+^ and, therefore, in the fading exponent a long component (τ_2_) is also observed. It can be seen that for both types of ceramics, the decay related to the shallow traps became longer with the increase in the graphene content and at the same time the longer component dropped instantly (Table 3). This may suggest that the presence of graphene in the ceramics promotes energy transfer from deeper traps, as they are released faster.

### 3.4. Impact of the Graphene Addition on the Thermoluminescence (TL) of Gd_2.994_Ce_0.006_Ga_3_Al_2_O_12_ and Gd_2.964_Ce_0.006_Dy_0.03_Ga_3_Al_2_O_12_ Ceramics

**Figure 8:**
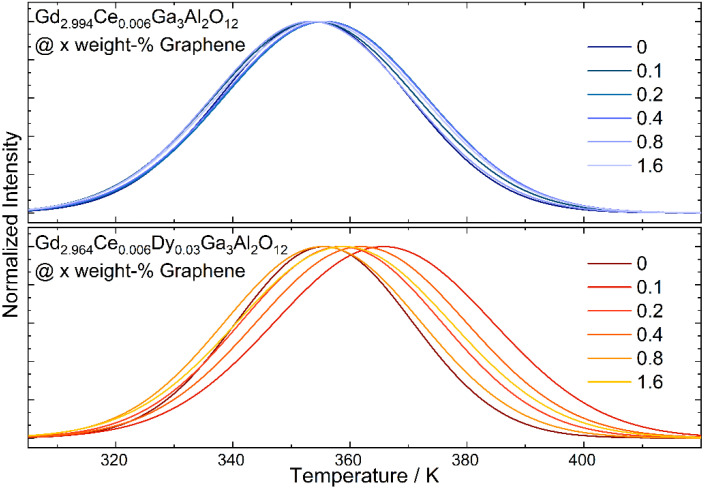
Normalized thermoluminescence (TL) glow curves registered for Gd_2.994_Ce_0.006_Ga_3_Al_2_O_12_ and Gd_2.964_Ce_0.006_Dy_0.03_Ga_3_Al_2_O_12_ ceramics with different graphene content.

The thermoluminescence glow curves were registered for ceramics with various additions of graphene flakes after irradiation by 450 nm laser diode (1 mW/cm^2^) for 5 min (Figure 8). All spectra were fitted using the OriginLab 2019b software with two peaks corresponding to the shallower and deeper traps. The position of the bands and its share in the overall thermoluminescence signal were extracted from the curves. The energy of the traps was calculated using the method proposed by Urbach [34]. Although this method is characterized by a great simplification, its use for all samples under the same conditions allows to estimate the course of changes taking place under the influence of the graphene amount in ceramics. The intensity of the thermoluminescence curve registered for ceramic co-doped with cerium and dysprosium is much higher than for the sample doped only with cerium and indicates a larger number of traps and electrons in them for samples co-doped with Dy. The intensity of TL curves measured for the ceramics with graphene decreases with the increase in graphene content. For a better comparison of the position and shape, all glow curves were normalized. It can be noticed that the position of the TL curve registered for solely Ce^3+^ doped ones shifts slightly towards higher temperatures, contrary to the glow curves registered for co-doped ceramics which are significantly changed. For the samples with the smallest amount of graphene, it is shifted toward a higher temperature, and then they move back to lower temperatures and again increase. A broadening of the band related to the extended trap distribution was also observed. It is assumed that the trap level is not regarded as a discrete energy level located in the band gap of the host material, but rather as a continuum of energy levels around a certain mean value [35]. The shift of the curves and its broadening is not only related to the energy of the traps but also to the share of each in the overall spectrum (Table 4). It can be seen that with an increase in graphene amount in the ceramic doped only with cerium, the changes are non-regular, but in the case of Dy co-doped samples, the share of deeper traps decreases. This may be an effect of a higher thermal conductivity and as a result faster thermal flow to the traps and hence the easier release of the carriers. The character of the traps associated with the graphene addition may be twofold. The graphene as a reduction agent may increase the oxygen-deficiency defects (oxygen vacancies) in the structure [36]. For the same reason, it can cause a change in the oxidation state of Ce and Dy ions, leading to the local charge mismatches and as a result the appearance of cation vacancies.

### 3.5. Thermal Conductivity of Gd_2.994_Ce_0.006_Ga_3_Al_2_O_12_ and Gd_2.964_Ce_0.006_Dy_0.03_Ga_3_Al_2_O_12_ Ceramics with Different Graphene Content

**Figure 9:**
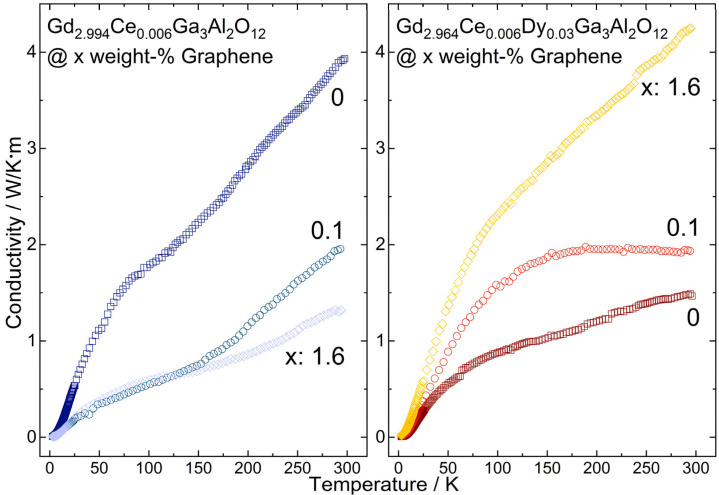
Thermal conductivity of Gd_2.994_Ce_0.006_Ga_3_Al_2_O_12_ and Gd_2.964_Ce_0.006_Dy_0.03_Ga_3_Al_2_O_12_ ceramics with different graphene content.

The impact of the graphene content in Gd_2.994_Ce_0.006_Ga_3_Al_2_O_12_ and Gd_2.964_Ce_0.006_Dy_0.03_Ga_3_Al_2_O_12_ ceramics on the thermal conductivity in the range from 2 K to 300 K was measured (Figure 9). For all investigated samples, the temperature dependence of thermal conductivity exhibited roughly the same character—disorder type, but with considerably higher values. The difference between particular ceramics: the Gd_2.994_Ce_0.006_Ga_3_Al_2_O_12_ and Gd_2.964_Ce_0.006_Dy_0.03_Ga_3_Al_2_O_12_ is clearly visible. The dysprosium addition to the ceramic structure lowers the thermal conductivity by two and a half, from almost 4 W/mK to 1.5 W/mK at room temperature. The reduction in the thermal conductivity value is caused by the increased number of scattering centers resulting from the higher amount of dopant in the structure. The higher the dopant amount, the lower the effective thermal conductivity value [37]. The mechanism changes when introducing into the material a substance of much higher thermal conductivity such as graphene in our case. The situation considered in this paper is twofold. For Gd_2.994_Ce_0.006_Ga_3_Al_2_O_12_, the addition of graphene lowers the thermal conductivity—implying that instead of improving the heat transfer in the structure graphene locates itself creating additional scattering sources. Just 0.1% of graphene reduced the heat transfer by half at room temperature, while the highest investigated graphene addition (1.6%) limits the thermal conductivity by three @RT. Below 140 K the difference between different graphene concentration on thermal conductivity is negligible. An entirely opposite process is observed for the Gd_2.964_Ce_0.006_Dy_0.03_Ga_3_Al_2_O_12_ sample. For the cerium/dysprosium co-doped case, adding the graphene enhances the heat flow as desired. Graphene acts here as a catalysator. The 0.1% graphene addition results in a third increase in thermal conductivity from 1.5 W/mK up to 2 W/mK at room temperature. The highest graphene addition (1.6%) lifts the thermal conductivity to 4.25 W/mK. The examined ceramics have demonstrated that improvement of heat flow cannot be straightforwardly achieved by just a graphene addition. The most important factor is the way that the graphene is incorporated into the matrix structure, and as a result, if it acts as a scatterer or carrier of heat.

### 3.6. Impact of the Graphene Addition on the Persistent Luminescence Mechanism of Gd_2.994_Ce_0.006_Ga_3_Al_2_O_12_ and Gd_2.964_Ce_0.006_Dy_0.03_Ga_3_Al_2_O_12_ Ceramics

**Figure 10:**
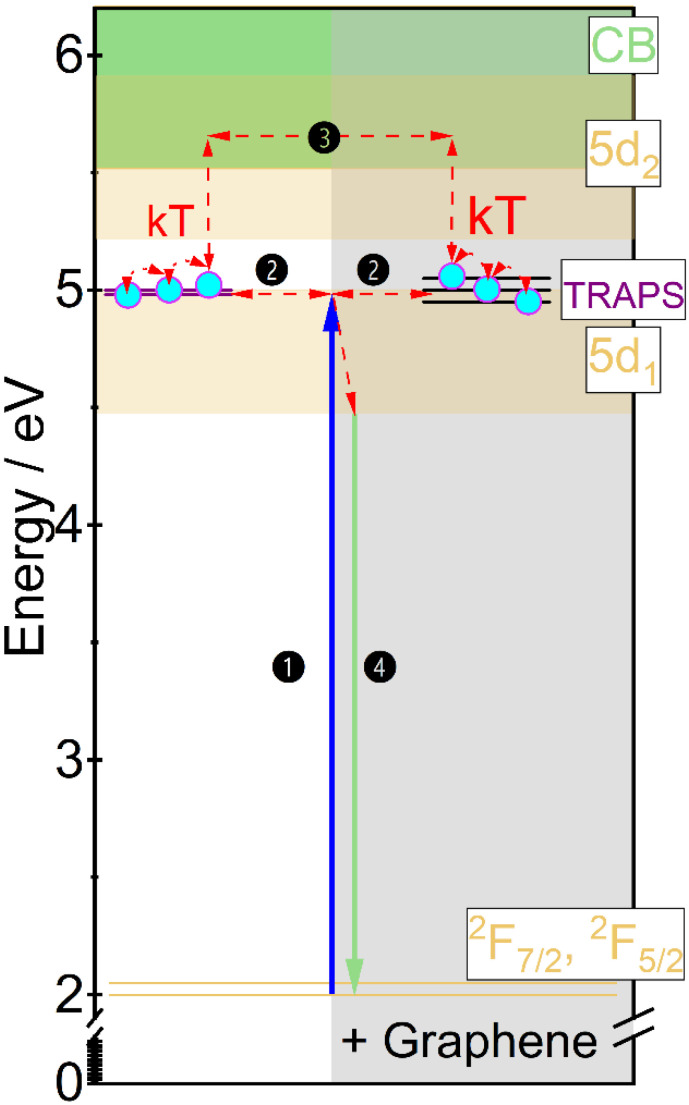
Mechanism of persistent luminescence proposed for the Gd_2.994_Ce_0.006_Ga_3_Al_2_O_12_ and Gd_2.964_Ce_0.006_Dy_0.03_Ga_3_Al_2_O_12_ ceramics with graphene addition.

The mechanism of persistent luminescence is proposed by adopting the energy transfer diagram already described by us [32] supplemented with changes induced by the addition of graphene (Figure 10). The position of 5d-4f levels was determined from the spectroscopic measurements and the position of the traps from the TL spectra. The changes in the mechanism are mainly associated with the change of thermal conductivity and lanthanide ions oxidation state after graphene addition. The process of traps charging is started under 455 nm irradiation. The electrons are excited from the ground state to the 5d level (1). Then, part of the electrons immediately recombines in the form of a photon giving conventional yellowish luminescence. If near the luminescent center an energy trap (defect) is located, part of electrons may be transferred through a tunnelling process to them [38] (2). As Gd_3_Ga_3_Al_2_O_12_ matrix has relatively small activation energy [39] the remaining electrons are transferred to the conduction band (CB) and through it are transported to the traps (3). In the ceramics where graphene was added, the traps have broader distribution, what simplifies electrons catching and releasing. The discharging process is started just after ceasing irradiation. The electrons captured in the traps may be released in two ways. One is using the same tunneling process (2) and pumping excited states of nearest luminescent center. The second is a transfer of the electrons to the 5d level of Ce^3+^ through CB (4). As the energy of the traps is relatively small (below 1 eV), the captured electrons may already be released at room temperature and due to improved thermal conductivity and greater traps distribution, it is also easier to release electrons from deeper traps. When the charge carriers are released, they move back to the emission center and then to the ground state giving persistent luminescence (4). As the charge carriers are gradually released from trapping centers, the persistent luminescence may take up to tens of minutes.

## 4. Conclusions

The influence of graphene addition on the persistent luminescence of the Gd_2.994_Ce_0.006_Ga_3_Al_2_O_12_ and Gd_2.964_Ce_0.006_Dy_0.03_Ga_3_Al_2_O_12_ ceramics was investigated. The XRD measurements show that in the ceramics containing graphene, the structure is maintained, but small changes in the crystallographic environment of luminescent ions may appear. SEM and EDS maps and their analysis show that the graphene added to the ceramics does not form a uniform network, which in some cases may hinder the interpretation of the results. In the ceramics excitation spectra, an increase in the splitting of the 5d states (Δ_21_) with the changing content of graphene can be observed, due to displacement from the cubic polyhedron and an increase in disorder in the environment of Ce^3+^ ions. The fading time of the persistent luminescence shows that after graphene addition, the shallower traps are emptied slower, while carriers from deeper traps are released faster, which shows that the presence of graphene mainly affects the traps with higher energy. These observations are confirmed by thermoluminescence measurements, where a shift of the glow curve towards higher temperatures (higher energies) is observed. The relationship between the depth of the traps, the presence of the Dy co-dopant and the changes observed in persistent luminescence are related to the increase in thermal conductivity after the addition of graphene, which was confirmed by thermal transport measurements. It can be concluded that the addition of graphene can improve the process of released electrons from deeper traps and have an influence on persistent luminescence, mainly in the materials where the charge carriers binding traps are deeper. However, for the best effect, low concentrations of graphene (about 0.1–0.2% by weight) and its homogeneous distribution between the grains should be preserved.

## Figures and Tables

**Table 1:** Position of the 5d levels, their full width and half maximum (FWHM) and the distance between 5d_1_ and 5d_2_ states (Δ_21_) calculated from excitation spectra of Gd_2.994_Ce_0.006_Ga_3_Al_2_O_12_ and Gd_2.964_Ce_0.006_Dy_0.03_Ga_3_Al_2_O_12_ ceramics with different graphene content.

Graphene Content	5d_2_ Maximum	FWHM (5d_2_)	5d_1_ Maximum	FWHM (5d_1_)	Δ_21_
Weight-%	cm^−1^				
**Gd_2.994_Ce_0.006_Ga_3_Al_2_O_12_**					
0	28,184	1874	22,490	2848	5694
0.1	29,137	1939	22,662	2229	6475
0.2	29,129	1948	22,656	2226	6473
0.4	29,165	1978	22,679	2180	6486
0.8	29,205	2049	22,702	2183	6503
1.6	29,247	2096	22,735	2207	6512
**Gd_2.964_Ce_0.006_Dy_0.03_Ga_3_Al_2_O_12_**					
0	28,292	1595	22,500	2973	5792
0.1	29,115	1873	22,674	2218	6441
0.2	29,129	1853	22,674	2193	6455
0.4	29,159	1866	22,691	2152	6468
0.8	29,260	2042	22,750	2116	6510
1.6	29,582	2830	22,653	2761	6929

**Table 2:** The maximum intensity position of persistent luminescence spectra and their FWHM registered for Gd_2.994_Ce_0.006_Ga_3_Al_2_O_12_ and Gd_2.964_Ce_0.006_Dy_0.03_Ga_3_Al_2_O_12_ ceramics with different graphene content.

Graphene Content	l_max_	FWHM
Weight-%	cm^−1^	
**Gd_2.994_Ce_0.006_Ga_3_Al_2_O_12_**		
0	18,156	2965
0.1	18,337	2766
0.2	18,397	2721
0.4	18,353	2789
0.8	18,385	2717
1.6	18,407	2788
**Gd_2.964_Ce_0.006_Dy_0.03_Ga_3_Al_2_O_12_**		
0	18,243	2699
0.1	18,294	2828
0.2	18,363	2855
0.4	18,393	2825
0.8	18,396	2759
1.6	18,382	2717

**Table 3:** Average fading time of persistent luminescence observed in Gd_2.994_Ce_0.006_Ga_3_Al_2_O_12_ and Gd_2.964_Ce_0.006_Dy_0.03_Ga_3_Al_2_O_12_ ceramics.

Graphene Content	τ_1_	τ_2_
Weight-%	s	
**Gd_2.994_Ce_0.006_Ga_3_Al_2_O_12_**		
0	49	212
0.1	38	148
0.2	40	126
0.4	42	159
0.8	52	107
1.6	60	89
**Gd_2.964_Ce_0.006_Dy_0.03_Ga_3_Al_2_O_12_**		
0	49	221
0.1	54	222
0.2	52	211
0.4	60	60
0.8	58	57
1.6	62	60

**Table 4:** TL glow curve maxima and energy of the traps calculated for Gd_2.994_Ce_0.006_Ga_3_Al_2_O_12_ and Gd_2.964_Ce_0.006_Dy_0.03_Ga_3_Al_2_O_12_ ceramics with different graphene content.

Graphene Content	T_max_	E	Share
Weight-%	K	eV	%
**Gd_2.994_Ce_0.006_Ga_3_Al_2_O_12_**			
0	369	0.74	44.9
	379	0.76	55.1
0.1	372	0.74	40.0
	379	0.76	60.0
0.2	370	0.74	41.3
	381	0.76	58.7
0.4	373	0.75	51.5
	381	0.76	48.5
0.8	373	0.75	43.9
	378	0.76	56.1
1.6	372	0.74	48.6
	381	0.76	51.4
**Gd_2.964_Ce_0.006_Dy_0.03_Ga_3_Al_2_O_12_**			
0	372	0.74	44.6
	381	0.76	55.4
0.1	376	0.75	46.9
	392	0.78	53.1
0.2	372	0.74	44.1
	384	0.77	55.9
0.4	375	0.75	45.4
	388	0.78	54.6
0.8	371	0.74	50.8
	382	0.76	49.2
1.6	376	0.75	52.8
	385	0.77	47.2

## Data Availability

The data presented in this manuscript and Supplementary Materials.

## Supplementary Materials

The following supporting information can be downloaded at: https://www.mdpi.com/article/10.3390/ma15072606/s1, Figure S1: XRD of commercial single layer graphene flakes; Figure S2: Raman spectrum of commercial single layer graphene flakes; Figure S3: TEM image of commercial single layer graphene flake.
