# Supplementary material for: Effect of Graphene Addition on the Thermal and Persistent Luminescence Properties of Gd2.994Ce0.006Ga3Al2O12 and Gd2.964Ce0.006Dy0.03Ga3Al2O12 Ceramics
Source: Materials (Basel). 2022 Apr 1;15(7):2606. doi: 10.3390/ma15072606 (PMC9000643; doi:10.3390/ma15072606)
Supplement: Supplementary file 1 [file materials-15-02606-s001.zip › materials-1652154-supplementary.pdf]

Supplementary Materials

Effect of Graphene Addition on the Thermal and Persistent Luminescence Properties of $Gd_{2.994}Ce_{0.006}Ga_3Al_2O_{12}$ and $Gd_{2.964}Ce_{0.006}Dy_{0.03}Ga_3Al_2O_{12}$ Ceramics

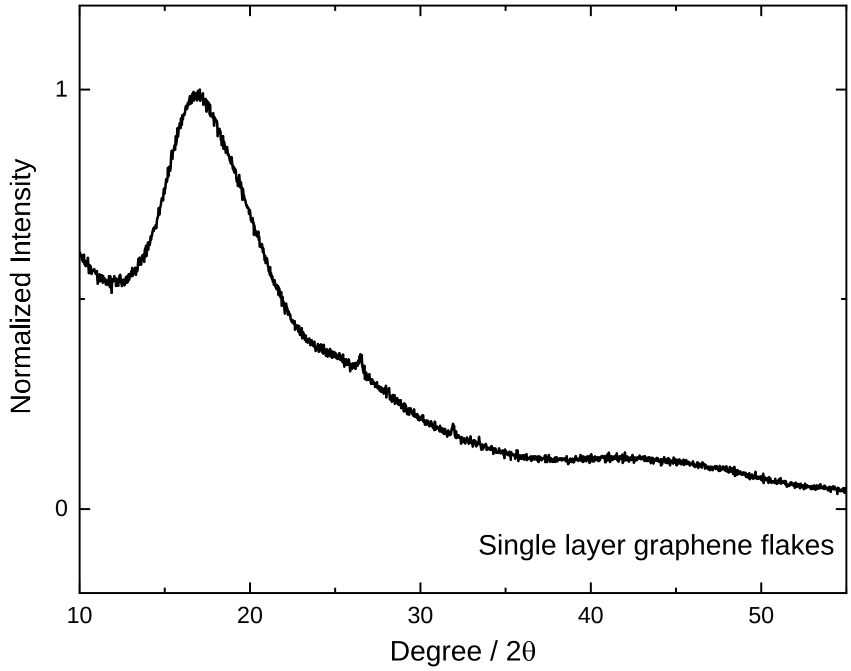

Figure S1. XRD of commercial single layer graphene flakes.

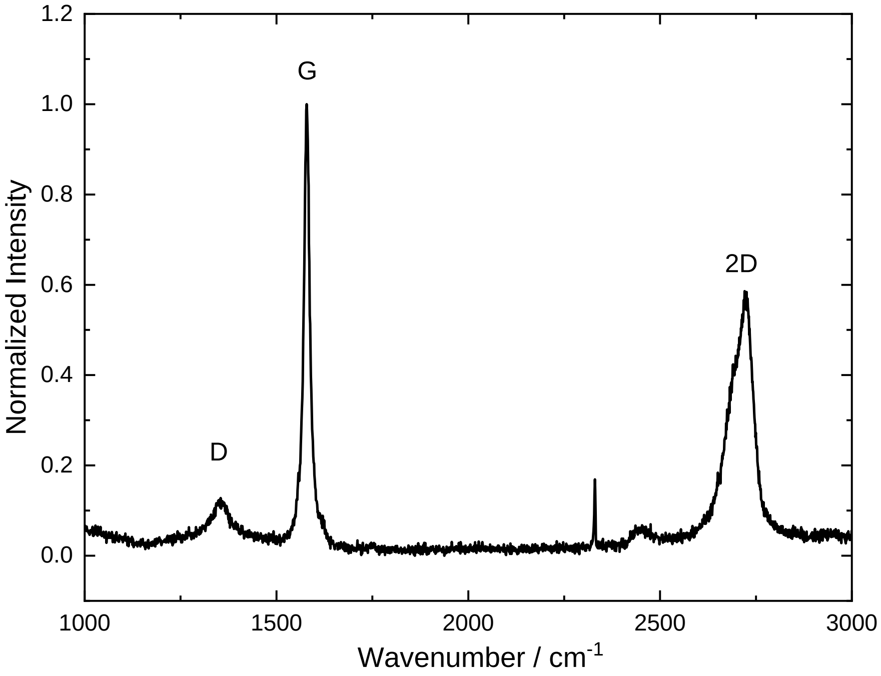

Figure S2. Raman spectrum of commercial single layer graphene flakes.

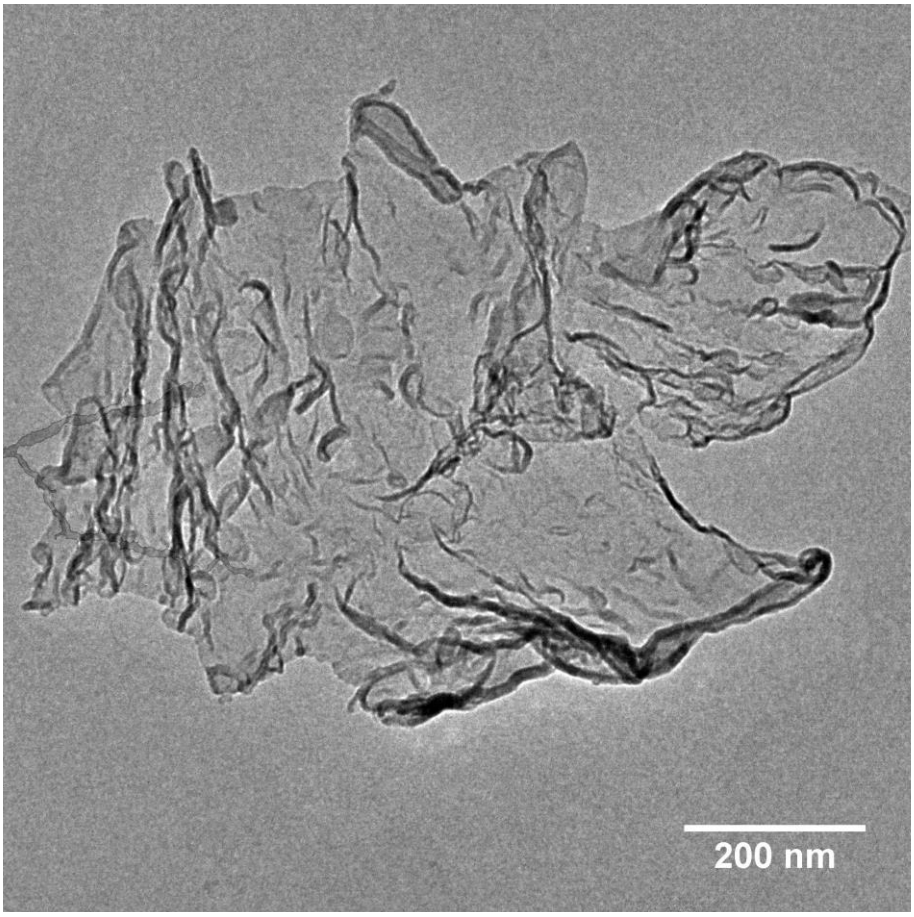

Figure S3. TEM image of commercial single layer graphene flake.
